# Microwave imaging for neoadjuvant chemotherapy monitoring: initial clinical experience

**DOI:** 10.1186/bcr3418

**Published:** 2013-04-24

**Authors:** Paul M Meaney, Peter A Kaufman, Lori S Muffly, Michael Click, Stephen P Poplack, Wendy A Wells, Gary N Schwartz, Roberta M di Florio-Alexander, Tor D Tosteson, Zhongze Li, Shireen D Geimer, Margaret W Fanning, Tian Zhou, Neil R Epstein, Keith D Paulsen

**Affiliations:** 1 Thayer School of Engineering, Dartmouth College, 14 Engineering Dr., Hanover, NH 03755, USA; 2 Oncology, Dartmouth-Hitchcock Medical Center, 1 Medical Center Dr., Lebanon, NH 03756, USA; 3 Radiology, Dartmouth-Hitchcock Medical Center, 1 Medical Center Dr., Lebanon, NH 03756, USA; 4 Pathology, Dartmouth-Hitchcock Medical Center, 1 Medical Center Dr., Lebanon, NH 03756, USA; 5 Biostatistics Shared Resource, Dartmouth-Hitchcock Medical Center, 1 Medical Center Dr., Lebanon, NH 03756, USA; 6 Radiology, Geisel School of Medicine, Dartmouth College, 74 College St., Hanover, NH 03755, USA; 7 60 Cobb Hill Rd., Hartland, VT 05048, USA; 8 Engineering, Kuang-Chi Institute of Advanced Technology, 29 Nanhuan Rd., Shenzhen, Guangdong, 518057, China

## Abstract

**Introduction:**

Microwave tomography recovers images of tissue dielectric properties, which appear to be specific for breast cancer, with low-cost technology that does not present an exposure risk, suggesting the modality may be a good candidate for monitoring neoadjuvant chemotherapy.

**Methods:**

Eight patients undergoing neoadjuvant chemotherapy for locally advanced breast cancer were imaged longitudinally five to eight times during the course of treatment. At the start of therapy, regions of interest (ROIs) were identified from contrast-enhanced magnetic resonance imaging studies. During subsequent microwave examinations, subjects were positioned with their breasts pendant in a coupling fluid and surrounded by an immersed antenna array. Microwave property values were extracted from the ROIs through an automated procedure and statistical analyses were performed to assess short term (30 days) and longer term (four to six months) dielectric property changes.

**Results:**

Two patient cases (one complete and one partial response) are presented in detail and demonstrate changes in microwave properties commensurate with the degree of treatment response observed pathologically. Normalized mean conductivity in ROIs from patients with complete pathological responses was significantly different from that of partial responders (*P *value = 0.004). In addition, the normalized conductivity measure also correlated well with complete pathological response at 30 days (*P *value = 0.002).

**Conclusions:**

These preliminary findings suggest that both early and late conductivity property changes correlate well with overall treatment response to neoadjuvant therapy in locally advanced breast cancer. This result is consistent with earlier clinical outcomes that lesion conductivity is specific to differentiating breast cancer from benign lesions and normal tissue.

## Introduction

Neoadjuvant chemotherapy (NCT) for breast cancer has become an increasingly important treatment option [1] that offers potential therapeutic advantages. Various clinical trials have demonstrated that treatment response, including tumor shrinkage, leads to substantial downstaging of disease which in turn allows for increased use of more limited surgery [1]. In such cases, breast conservation strategies such as lumpectomies and radiotherapy can be used instead of more comprehensive radical mastectomies [2]. By monitoring the patient's response to systemic therapy before tumor resection, prediction of longer term prognosis or identification of the need for additional or alternative treatment may be possible. Data have suggested that patients receiving neoadjuvant therapy with pathologic complete response have better survival outcomes [1,3].

While NCT is emerging as a promising treatment strategy for locally advanced breast cancer, deploying monitoring methods during the course of therapy is important for making further advances. Magnetic resonance (MR) and fluorodeoxyglucose positron emission tomography (FDG PET) have been evaluated in several clinical trials and have proven useful in this setting, but both also come at considerable cost and inconvenience [3-8]. Conventional breast imaging modalities, such as ultrasound and mammography, have been disappointing in detecting the extent of residual disease during treatment [9]. Studies have also been conducted using Doppler ultrasonography, diffuse optical spectroscopy and tomography and scintimammography [10-13]. Assessing tumor shrinkage by clinical breast examination is possible as well but is poorly correlated with treatment response [1].

Microwave imaging is in the early stages of clinical evaluation and most of the work has focused on breast tumor detection. Impressive technical developments based on active microwave radar concepts have been reported [14-18]. Passive microwave radiometry has also been investigated [19-21]. However, only a modest number of clinical microwave tomographic (MT) breast examinations have been conducted [22-24]. Our early clinical experience with MT has demonstrated statistically significant differences in the electrical conductivity of breast tumors greater than 1 cm in diameter relative to the normal breast and other benign abnormalities [25]. We have also shown strong associations between whole breast radiographic density designations as well as focal patterns of dense fibroglandular tissue and the imaged microwave property distributions [26].

We are currently evaluating women undergoing NCT for the treatment of locally-advanced breast cancer with MT performed at regular intervals from the start of treatment to the time of surgery. In the following sections, we describe the methods used in the study related to patient recruiting, imaging modalities, pathology and image analysis. In the Results section, we examine two cases in detail (one complete responder and a second non-responder) to provide an appreciation of the information contained in the images and their progression during treatment. In addition, we also include a representative sequence of images from the contralateral breast for one patient during the course of treatment as an example of the more limited changes observed in the non-cancerous breast. Finally, we statistically analyze the results for the eight patients in this study.

## Materials and methods

### Patient recruiting

The microwave imaging study was Health Insurance Portability and Accountability Act (HIPAA) compliant and approved by the institutional review board (IRB) at Dartmouth. These experiments comply with the current laws of the United States of America. All patients provided written informed consent and were compensated for their participation. Eight women enrolled in a pilot study, successfully completed their treatment and corresponding series of MT examinations. These patients proceed through the prescribed chemotherapy regimen until surgery as part of standard-of-care breast cancer management. In most cases, MR images of both breasts were acquired just prior to therapy and again after treatment but before surgery (one case was followed with ultrasound examinations). After surgery, all breast tissue was analyzed in Surgical Pathology at Dartmouth-Hitchcock Medical Center (DHMC) to document the extent of treatment response. Table 1 summarizes the patient information for the subjects enrolled in this pilot stage of our study.

**Table 1:** Summary of patient data

Study Number	Breast Density	Size (cm)	Pathology	Exams	Treatment	Radiographic Response	Pathologic Response
1	SC	7 × 6.5	IDC, ER+/PR-/HER2neu+	7	AC^2^/paclitaxel trastuzumab	Yes (MRI)	pCR

2	HD	5 × 3	IDC, ER-/PR-/HER2neu-	7	paclitaxel tocosol	No substantial response (MRI)	pPR2

3	SC	4 × 4	IDC, ER-/PR-/HER2neu-	5	TAC^3^	Yes (MRI)	pCR

4	HD	5 × 5	IDC, ER+/PR+/HER2neu+	5	AC^2^/paclitaxel trastuzumab	Yes (MRI)	pCR

5	ED	8 × 7	IDC, ER+/PR+/HER2neu-	10	AC^2^/paclitaxel	No substantial response (MRI)	pPR4

6	HD	1 × 1	IDC, ER equivocal/PR-/HER2neu+	8	Taxol/FEC/Herceptin	Yes (MRI)	pCR

7	ED	10 × 9	IDC, ER-/PR-/HER2neu+	4	TAC^3^	Yes (MRI)	pPR1

8	HD	4 × 4	IDC, ER+/PR+/HER2neu+	5	TAC^3^	No substantial response (MRI)	pPR3

AC^2^, doxorubicin and cyclophosphamide; ED, extremely dense; ER, estrogen receptor; HD, heterogeneously dense; HER2-neu, human epidermal growth factor receptor 2; IDC, invasive ductal carcinoma; ILC, invasive lobular carcinoma; pPR1, near total treatment effect (< 10% of tumor remaining) [32]; pPR2, evidence of response to therapy but with 10% to 50% of tumor remaining [32]; pPR3, evidence of response to therapy but with > 50% < 90% of tumor remaining [32]; pPR4, minimal evidence of response to therapy with > 90% of tumor remaining [32]; PR, progesterone receptor; SC, scattered; TAC^3^, docetaxel, doxorubicin, and cyclophosphamide.

### Microwave Tomography

For each TM session, patients lie prone on a padded table with one breast pendant through an aperture into a coupling liquid. An array of monopole antennas with fixed radial positions on a 15.2 cm diameter circle are moved up from the base of the imaging tank into a position surrounding the breast close to the chestwall. Data are acquired over multiple frequencies (700 to 1,700 MHz in 200 MHz increments) with each antenna sequentially acting as the transmitter and the remaining complement serving as receivers for a total of 240 (16 transmitters × 15 receivers) measurements at each frequency. The antennas are subsequently moved to six lower positions in 1 cm increments (or less depending on the overall breast length) where the data acquisition sequence is repeated to provide coverage of the breast. After data acquisition is completed on one breast, the patient is repositioned and the contralateral breast is imaged under the same protocol for a total examination time of approximately 15 minutes (approximately 5 minutes of data acquisition time per breast and 5 minutes for breast positioning).

We utilized either 80% or 86% glycerin coupling baths for patient examinations. At the first pre-therapy imaging session, baseline data were acquired with both baths for each patient. We determined the optimal bath for a given subject by evaluating the measured data and image quality from both acquisitions [26]. Given the variability of the average microwave properties of the breast, especially with respect to its density and the patient's age, we compared the recovered images empirically on a patient-by-patient basis in order to determine the best mixture for future examinations [24]. Once the bath of choice was established, the liquid was recycled and used only for the subsequent examinations of that individual patient.

Permittivity and conductivity images were reconstructed post-examination for associated frequencies and antenna positions. Each image was processed in less than two minutes on a Dell Blade workstation using our two-dimensional finite difference time domain (FDTD)-based algorithm which has been described in detail elsewhere [27-30].

### Image analysis

**Figure 1:**
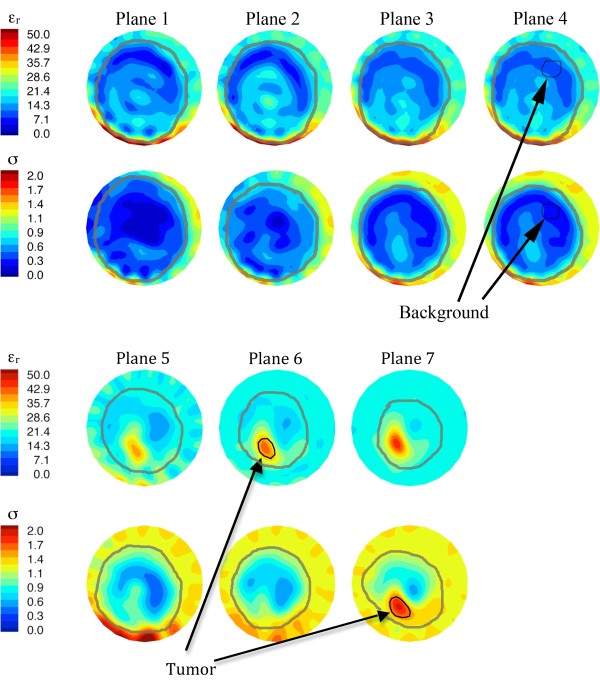
**1300 MHz permittivity (top row) and conductivity (bottom row) images for all 7 planes**. Breast perimeter, and tumor ROI and background zones used in the analysis are indicated. ROI, region of interest.

Regions of interest (ROIs) were determined for each set of permittivity and conductivity images at 1,300 MHz which proved to be the most reliable and highest resolution images obtained over all patients and examination dates. Each ROI was selected at one array position based on knowledge of the central tumor location from information provided by the attending oncologist with support from the study radiologists - both in terms of distance from the chestwall and spatially within the anatomically coronal plane. Given that breast positioning from examination to examination was not identical, and that the breast may change size over the course of therapy due to structural and compositional changes from treatment, care was exercised to align the ROI plane between sessions. Similarly-sized regions were also applied to reduce the comparison bias between examinations. The same process was performed to define representative non-tumor tissue ROIs for generating the background metrics used in the analysis. A graphical user interface was used to identify each zone and compute the microwave property averages and associated standard deviations automatically. Figure 1 shows a representative example of a set of breast images used in the analysis. The ROIs resulted from planes where the tumors were dominant in the permittivity and conductivity images. The background zones were selected from planes farther away from the dominant tumor cross-sections to minimize any influences of the elevated tumor properties on the background estimate that can occur from the smoothing characteristics of the image regularization process. In addition, because the normal sections of the breast contain zones of adipose and fibroglandular tissue, and the properties of the latter are known to vary considerably, we chose background regions consisting primarily of adipose tissue (as demonstrated by their very low property values) as a stable and consistent baseline for the duration of the study [31].

### Clinical (MR) imaging

MR breast images were acquired in a 1.5T scanner (GE Signa, GE Healthcare, Waukesha, WI, USA) for standard clinical indications or a 3T (Philips Achieva, Philips Healthcare, Andover, MA, USA) system for research studies. Dedicated breast coils were used with sub-millimeter in-plane resolution and slice thicknesses of < 3 mm (T1-weighted) and < 5 mm (T2-weighted). T1 and T2 scans provided detailed maps of tissue structure which were used to differentiate breast tissue types. Three dimensional gradient echo T1-weighted sequences with chemical fat suppression were performed prior to and at one to two minute intervals following the injection of a bolus of contrast (Gadodiamide, Omniscan, GE Healthcare) of 0.1 mmol per kilogram of body weight to identify suspicious enhancement foci. The first post-contrast scan was initiated 40 seconds after injection. Subtraction images were generated with the pre-contrast gradient echo T1 image as the reference to assess the Gd washout behavior.

### Pathology

Standard surgical pathology analysis of the excised tissues was used to assess subject outcomes. The subjects compared in this pilot study underwent surgical mastectomy [32]. The mastectomy specimens were sectioned fresh, medial to lateral, in the sagittal plane. Each tissue slice was examined grossly and photographed for image correlation. Areas of treatment response, residual tumor and normal breast were sampled and processed for microscopic analysis per standard laboratory protocols (formalin fixation, dehydration, paraffin-embedding, 4 micron sectioning and H & E stain). Complete pathologic response (pCR) was defined as a complete absence pathologically of cancer in the breast after extensive sampling.

## Results and discussion

### Case I - complete response

The first patient had a locally advanced cancer on the right side. At diagnosis, the right breast mammogram showed linear and pleomorphic micro-calcifications in a segmental distribution over the lower-central and entire lower-inner gradient while also demonstrating skin thickening in overlying zones. The tumor measured 6.5 × 3.7 × 7.1 cm by MRI. Biopsy demonstrated an intermediate grade invasive ductal carcinoma with a component of ductal carcinoma *in situ *(DCIS), estrogen receptor positive (ER(+)), progesterone receptor negative (PR(-)), human epidermal growth factor receptor-2 (HER-2)/neu(+) (HER-2:CEP-17 14.2). The patient was treated with four cycles of doxorubicin and cyclophosphamide, followed by four cycles of weekly paclitaxel and traztuzumab (PT) and then underwent modified radical right mastectomy. Surgical pathology revealed a complete pathologic response.

**Figure 2:**
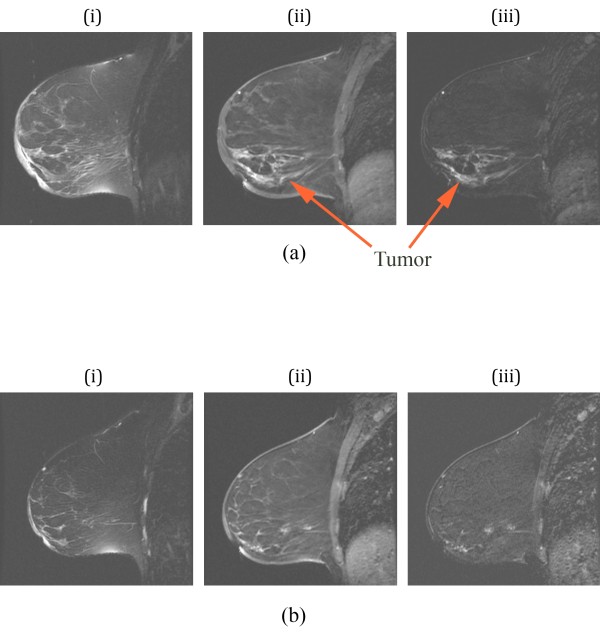
**Sagittal MR images of the right breast of a patient with a complete pathologic response: (a) images prior to therapy and (b) at day 85**. For each set, (i) is a T2-weighted image, (ii) is a contrast-enhanced image from a SPGR sequence, and (iii) is a subtraction image between (ii) and a pre-contrast baseline, respectively. MR, magnetic resonance; SPGR, spoiled gradient recalled.

Contrast-enhanced MRI was performed prior to treatment, at day 85, and at completion of neoadjuvant therapy prior to surgery. Figure 2a shows a sequence of corresponding sagittal images of the right breast prior to treatment consisting of (i) a baseline T2 sequence, (ii) a gadolinium enhancement image and (iii) a baseline subtraction image. The dominant feature reveals a large dispersed tumor in the lower portion of the breast extending from the nipple to the chestwall. Enhancement of lymph nodes in the upper right corner of the images is also evident. The contrast enhanced image exhibits significant skin thickening around most of the breast, but especially near the nipple. In addition, the T2 image shows substantial edema (because it does not enhance in the subtraction image) under the skin in the regions near the nipple which is due to the dermal lymphatics. The corresponding images at day 85 (Figure 2b) show enhancement in the same span from the nipple to the chestwall but at a reduced level, indicative of response at this intermediate point during treatment. By this time, the skin has also thinned considerably with a corresponding reduction in edema.

**Figure 3:**
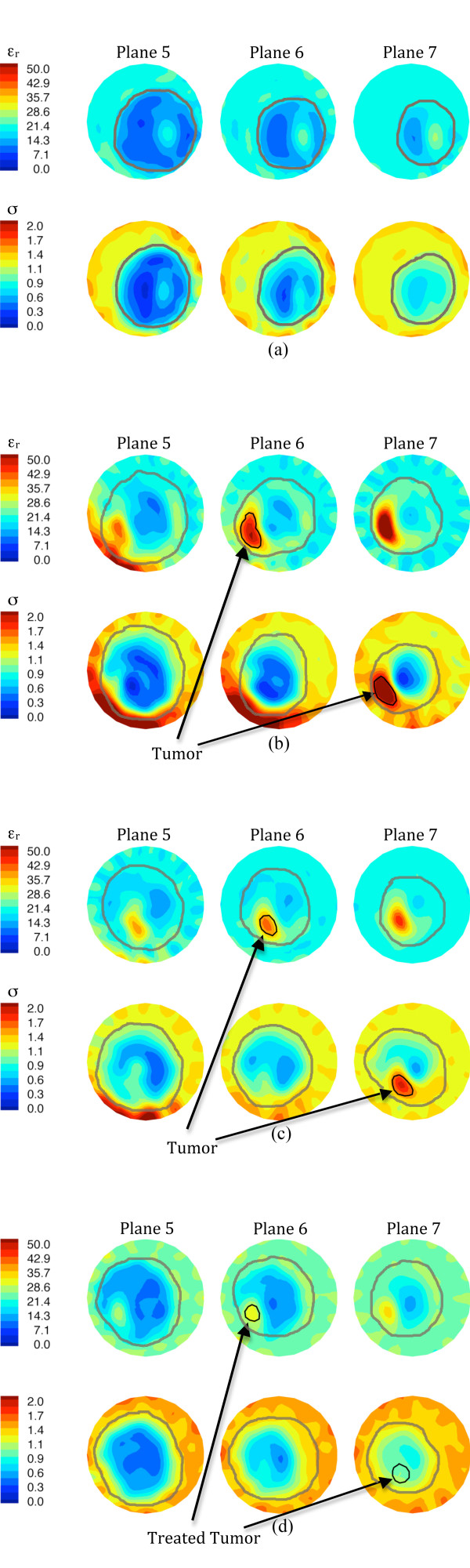
**1300 MHz microwave tomographic images**. Imaging planes five to seven are shown corresponding to the three closest planes to the nipple with the permittivity on the top row and conductivity on the bottom row. (a) Left (contralateral) breast prior to treatment, (b) right (ipsilateral) breast prior to treatment, (c) right breast 44 days into treatment, and (d) right breast immediately prior to surgery.

MT was conducted prior to the start of therapy, five other times during treatment and just prior to surgery. Figure 3 shows the recovered, *en face *permittivity and conductivity images for anatomically coronal planes 5, 6 and 7 (Plane 1 is closest to the chestwall and subsequent planes move away in 1 cm increments) for (a) the left (contralateral) breast before chemotherapy, (b) the right (ipsilateral) breast before chemotherapy, (c) the right breast at day 44, and (d) the right breast after therapy. For the left breast, the tissue boundary is readily evident and decreases in size from plane 5 to 7 as expected. Both permittivity (ε_r_) and conductivity (σ) images show the breast exhibiting relative low values with a somewhat elevated central region most likely associated with higher water-content fibroglandular tissue [31].

For the right (diseased) breast images prior to treatment, the coronal perimeters are more uneven with elevated property zones in the lower quadrants and increased properties on the breast boundary likely corresponding to the tumor, skin thickening and edema appearing in the MRI, respectively. At day 44 (Figure 3c) the baseline MT images have changed significantly and show: (1) improved delineation of the breast boundary, (2) diminished tumor size and property intensity, and (3) reduced property intensity along the lower breast edge. All three findings appear to correspond well with the clinical tumor size reduction, skin thinning and decrease in subdermal edema. At the end of treatment, the breast outline is even more regular than in the previous images, the recovered tumor enhancement is further reduced and no enhancement appears near the breast perimeter.

A significant reduction in the recovered dielectric properties occurred within the tumor region as treatment progressed. The post-surgical pathological evaluation confirmed a complete response. Larger areas of soft, grey-white tissue appear where the tumor had been treated. Microscopic analysis showed no signs of viable tumor with only stromal scarring, macrophages and inflammation in the treated areas. Dielectric probe measurements in pathology revealed that the treated tumor properties have not reverted back to values corresponding to fibroglandular zones in the contralateral breast as expected since the treated area is no longer representative of normal breast tissue.

**Figure 4:**
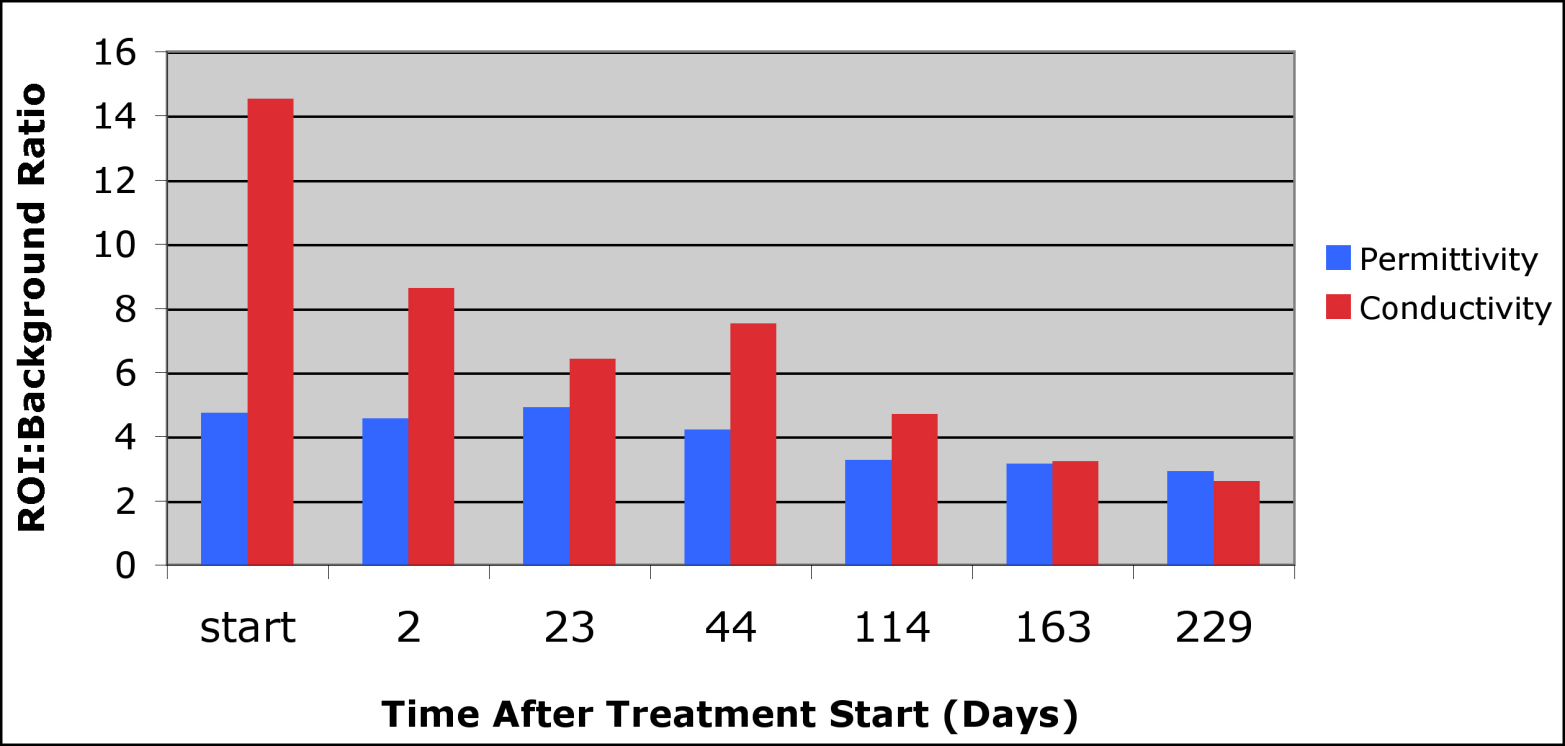
**Summary of the permittivity and conductivity region of interest (ROI) analysis for the MT imaging sessions from the start of treatment until just prior to surgery**. MT, microwave tomography.

Figure 4 summarizes the 1,300 MHz ROI:background property ratios as a function of time for all MT examinations during treatment of this patient. The permittivity ratio decreased somewhat unevenly and had a total reduction of 38% from the start to end of treatment. The ε_r _value did not drop below 89% of its initial level until the day 114 examination. The conductivity ratio exhibited a more dramatic and earlier reduction - 56% decrease after 23 days and 82% decrease after treatment completion, respectively, which is consistent with our previous clinical results that indicated the conductivity images are significantly more sensitive to the presence of tumor than their permittivity partners.

### Case II - non-responder

The second case was a woman who presented with locally advanced and metastatic breast cancer. Initial MRI showed a 4 × 2.5 × 4 cm irregular complex cystic mass in the upper outer quadrant of the left breast. Two smaller (1 cm) lesions were noted superior and anterior to the primary tumor. Core needle biopsy of the primary tumor demonstrated a high grade invasive ductal carcinoma with angiolymphatic invasion, weakly ER(+), PR(-), HER-2/neu(-) (HER-2:CEP-17 1.09). Left axillary lymph node biopsy was positive for metastatic disease. The patient was treated with four cycles of single agent weekly TOCOSOL paclitaxel (Eagle Pharmaceuticals, Belgrade, Serbia), followed by left simple mastectomy. Surgical pathology revealed a partial pathologic response with the treatment effect estimated as comprising > 50% but < 90% of the original tumor.

**Figure 5:**
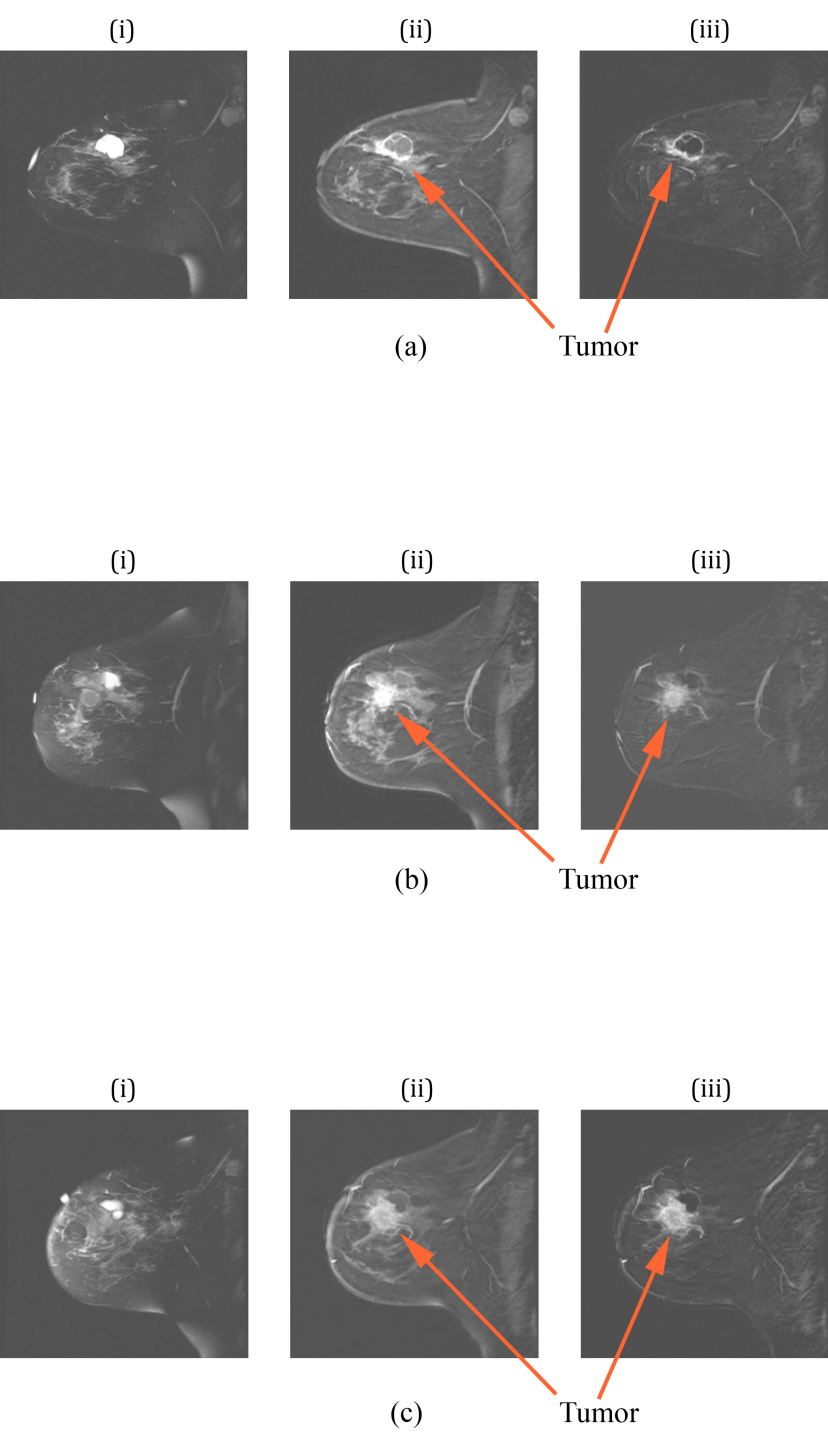
**Sagittal MR images of the left breast of a patient with pathologic non-response: (a) images prior to therapy, (b) images on day 52, and (c) images prior to surgery**. For each set, (i) is a T2-weighted image, (ii) is a contrast-enhanced image using an SPGR sequence, and (iii) is a subtraction image between (ii) and a baseline, respectively. MR, magnetic resonance; SPGR, spoiled gradient recalled.

MR images were obtained throughout neoadjuvant treatment and two weeks prior to mastectomy (day 85). Figure 5a shows a sequence of corresponding sagittal images of the left breast prior to treatment with (i) a T2 sequence, (ii) a gadolinium enhancement image and (iii) a baseline subtraction image. The pre-treatment MR images presented a large mass with in-plane gadolinium-enhancing dimensions of 4.7 × 2.6 cm and a secondary satellite (anterior) enhancement. A large water filled cavity (seroma resulting from an earlier biopsy) appeared directly adjacent to the tumor. Figure 5b shows the corresponding images for day 52 which exhibit a more pronounced tumor adjacent to the seroma (visible as a dark region in the subtraction image) with distinct spiculations radiating from the center. This MR set showed an enhancing zone of 3.5 × 4.3 cm in a similar area with possible progression anteriorly. The washout kinetics were similar to those of the first examination. A later study at day 85 indicates that the satellite lesion became more active in terms of its enhancement/rapid washout rate suggesting tumor progression in this area. By the time of the last MR (not shown), the gadolinium-enhancing zone had grown to 5.4 × 3.9 cm and showed washout kinetics indicative of malignant progression. This study suggested that the larger primary mass had merged with the secondary satellite focus evident in the first examination.

**Figure 6:**
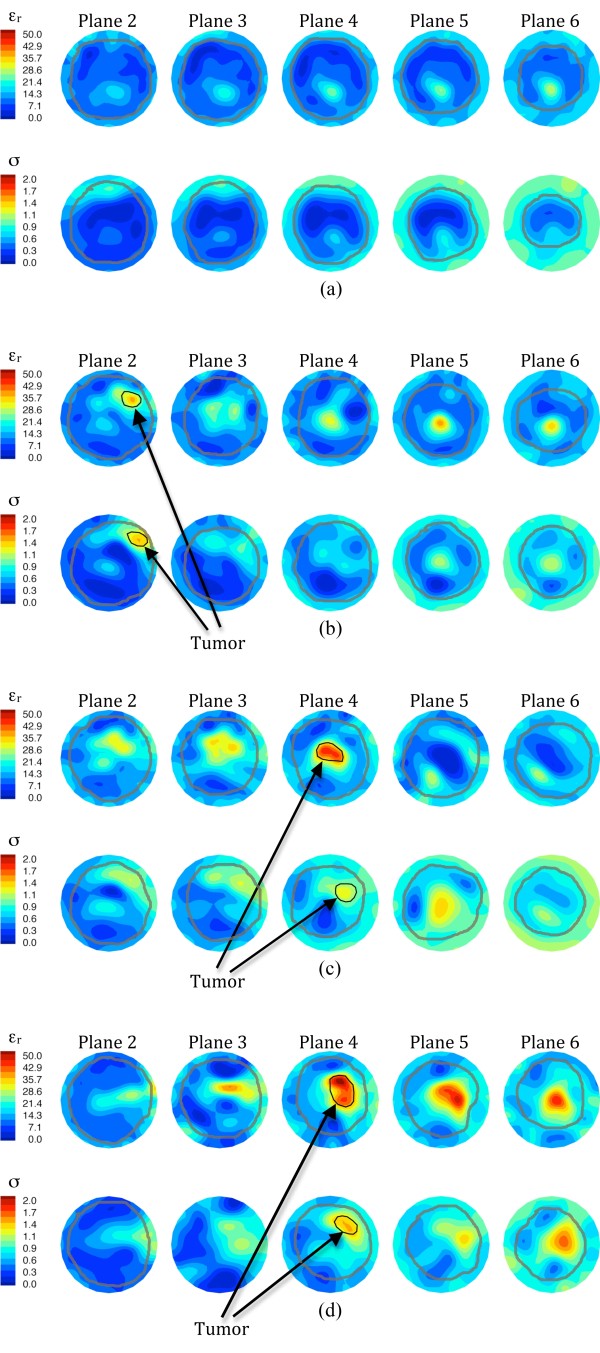
**1300 MHz microwave tomographic images**. Imaging planes two to six are shown corresponding to the five closest planes to the nipple with the permittivity on the top row and conductivity on the bottom row. (**a**) Right (contralateral) breast prior to treatment, (**b**) left (ipsilateral) breast prior to treatment, (**c**) left breast 57 days into treatment, and (**d**) left breast immediately prior to surgery.

MT was conducted prior to the start of therapy, at five times during treatment and just prior to surgery. Figure 6 shows the reconstructed images for planes two to six (1.0 cm increments) for (a) the right breast at the start of chemotherapy and for the left breast at (b) the start of chemotherapy, (c) day 28, and (d) just prior to surgery. The right breast images present relatively homogeneous dielectric properties in all planes, except for an elevated zone in the lower central to lower-right quadrant which probably corresponds to a concentration of fibroglandular tissue. As is typical with these images, the outline of the breast is evident and decreases in diameter as the planes progress towards the nipple. Some artifacts appear in plane two (closer to the chestwall), especially an unevenness in the breast permittivity perimeter and a slightly elevated zone in the upper-left edge of the conductivity image. These artifacts are fairly common occurrences in our images, especially for larger breasts and those with very high property contrast with the coupling bath, and have been noted previously in Meaney *et al. *[26,33].

For the left breast images, the outer boundaries appear more disrupted than in the contralateral breast and significant internal zones of both permittivity and conductivity enhancement are evident. Enhancement occurs in the upper-central to upper-right regions in planes two to four of the permittivity and planes two to five of the conductivity images at the first two imaging dates (pre-chemotherapy and day 52). This visibility over multiple planes is indicative of a large tumor. The enhanced area in plane four of the permittivity image from the second examination is much more intense (higher values) than the corresponding plane for the first examination and appears to corroborate the MR observation that the tumor was growing. The microwave images obtained from the last examination suggest that the tumor has expanded significantly in size with the appearance of substantial enhancement in both planes five and six. The level of enhancement in the conductivity images has also increased correspondingly. These results correlate well with the clinical observations that the tumor spread anteriorly towards a satellite location and that the primary and secondary tumors had merged by the time of surgery.

**Figure 7:**
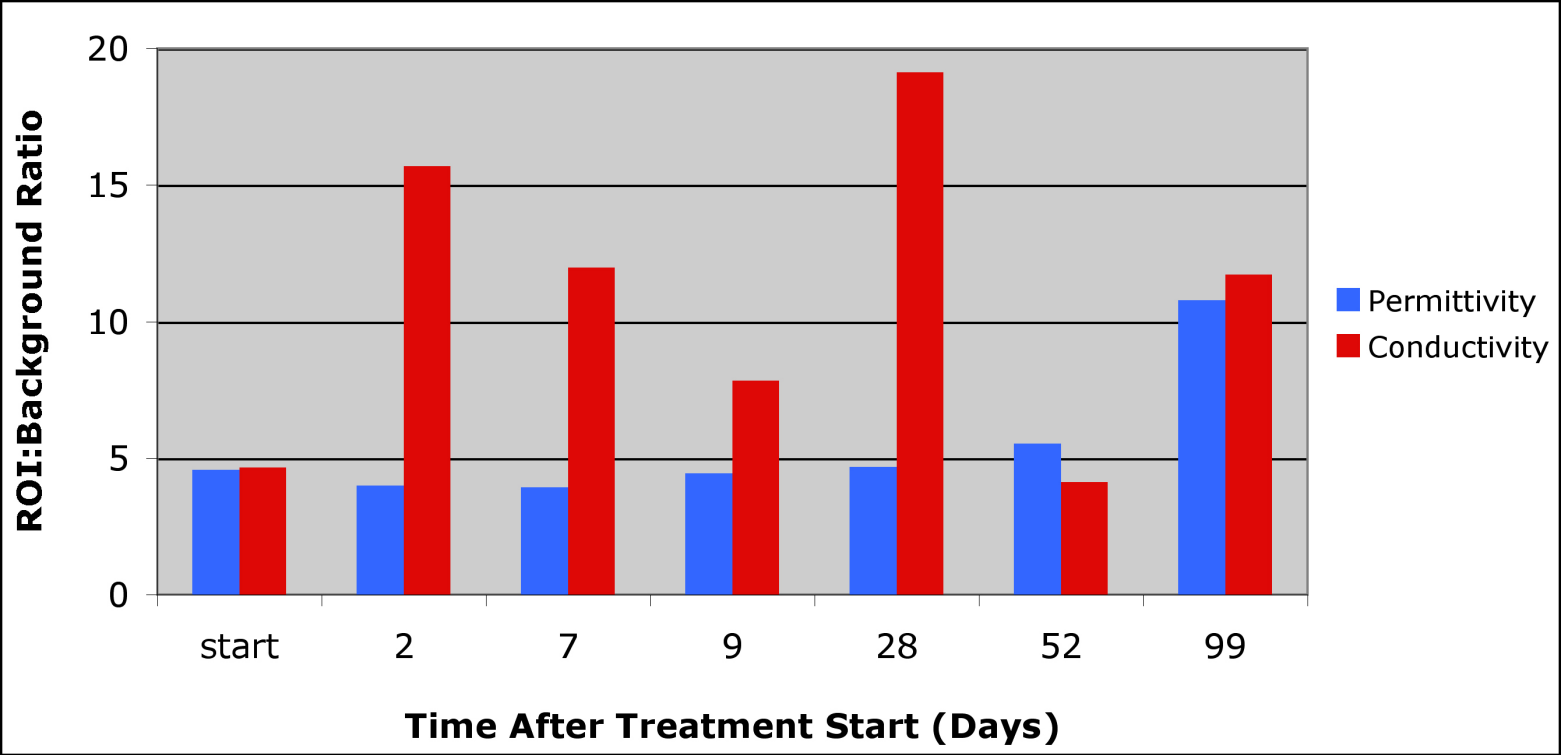
**Summary of the permittivity and conductivity region of interest (ROI) analysis for the MT imaging sessions from the start of treatment until just prior to surgery**. MT, microwave tomography.

Figure 7 summarizes the ROI:background ratios for both the permittivity and conductivity images as a function of time. In this case, the permittivity ROI:background values remain relatively constant but then increase dramatically towards the end of treatment. The corresponding conductivity ratios are much more uneven with sharp increases at day 2 and day 28. The uneven behavior may be due in part to the fact that the tumor did appear to partially respond in some areas (for example, during the first 21 days) but then expanded towards the anterior portion of the breast. Nonetheless, the ROI:background values did not decrease appreciably at any extended duration during treatment and are consistent with the pathological determination of non-response.

### Contralateral example

**Figure 8:**
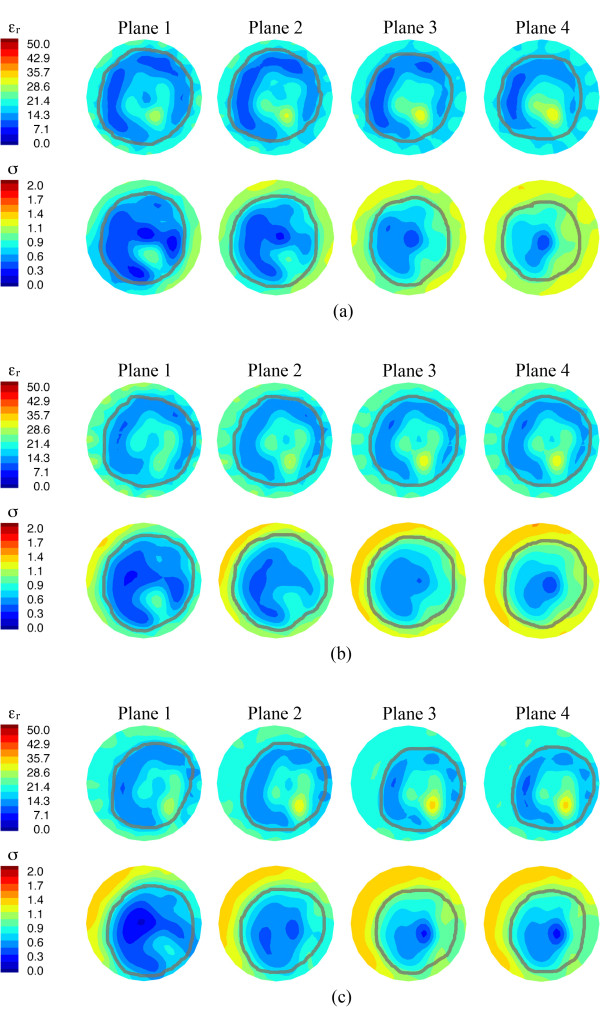
**1300 MHz microwave tomographic images of the contralateral breast for Patient 8**. Imaging planes one to four are shown corresponding to the four closest planes to the chest wall with the permittivity on the top and conductivity on the bottom row, respectively. (**a**) Prior to treatment, (**b**) day 33, and (**c**) day 62.

For comparison, we also collected data from the contralateral breast when possible (that is, examination time-permitting, patient not feeling ill, and so on) and show example images from planes one to four for Patient #8 at the first date, day 33 and day 62 after the start of treatment (Figure 8). In these images, the permittivity and conductivity outlines of the breast are evident in terms of a property gradient with the background coupling fluid. As the images progress from plane one to four, the overall breast diameter decreases - a feature that is more obvious in the conductivity results. In the permittivity images, an annulus of lower property tissue surrounds a more elevated zone in each of the four planes. This central region contains more fibroglandular tissue for which higher permittivity properties are expected relative to the surrounding adipose tissue. In the conductivity images, the first two planes also show elevated property zones towards the lower right quadrant; however, they begin to disappear in planes three and four as the overall size of the breast cross-section shrinks, and the increased fibroglandular tissue properties begin to blur with the higher properties of the surrounding coupling liquid. The images for all three examination dates are similar and do not suggest significant changes other than from minor shifting of the position of the breast within the field of view.

We were not always able to collect as much data from the contralateral breast as for the ipsilateral (diseased) side for multiple reasons: (a) several patients had ports inserted in the contralateral breast for drug infusion that precluded imaging, (b) multiple experimental imaging modalities were involved in the study, so the total examination time allotted to MT was limited, and (c) some patients were simply feeling too ill to participate in more than a single-sided breast examination.

### Summary of the eight patient study

**Figure 9:**
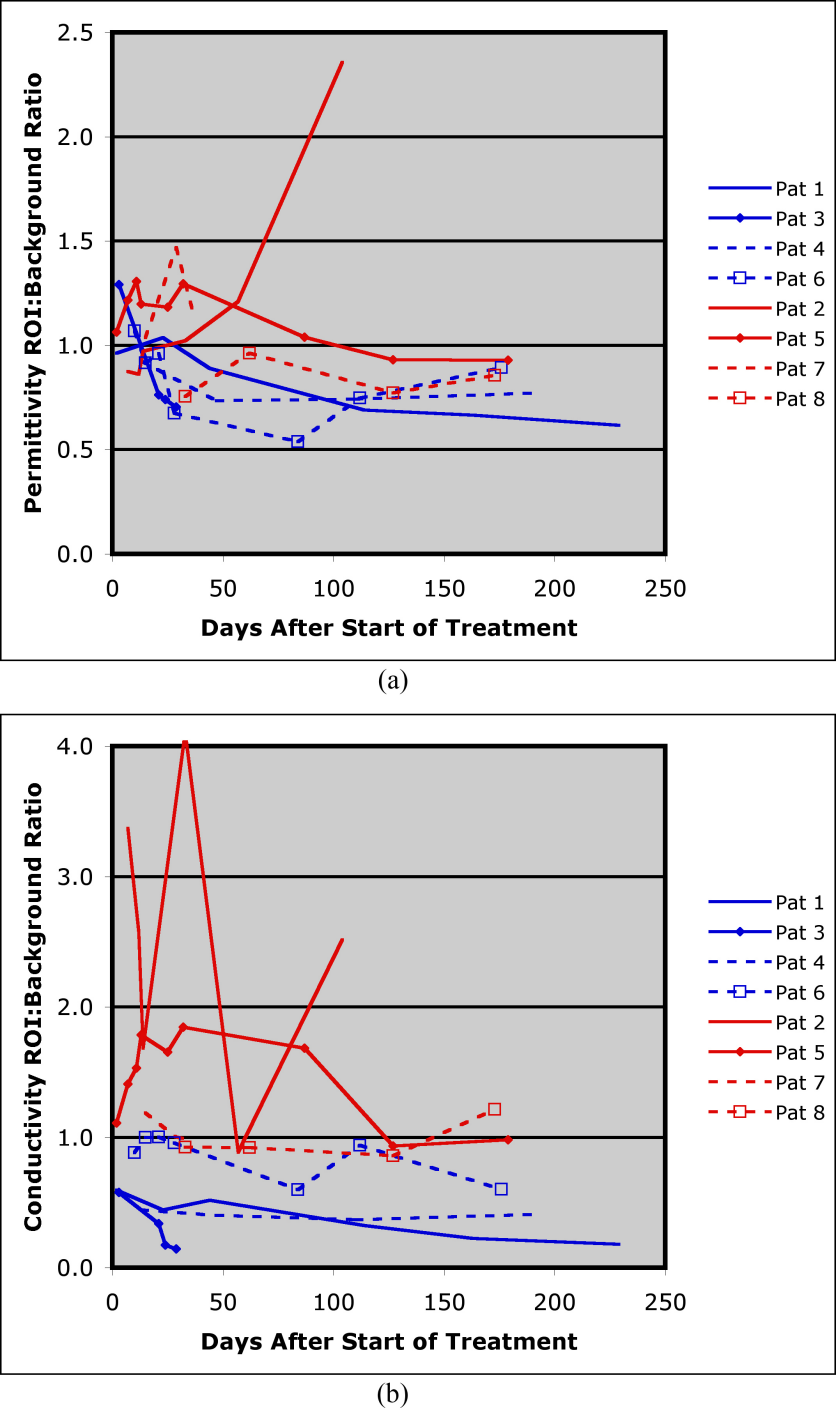
**Summary of the permittivity and conductivity region of interest (ROI) analysis for the MT imaging sessions for all patients during treatment**. MT, microwave tomography.

**Figure 10:**
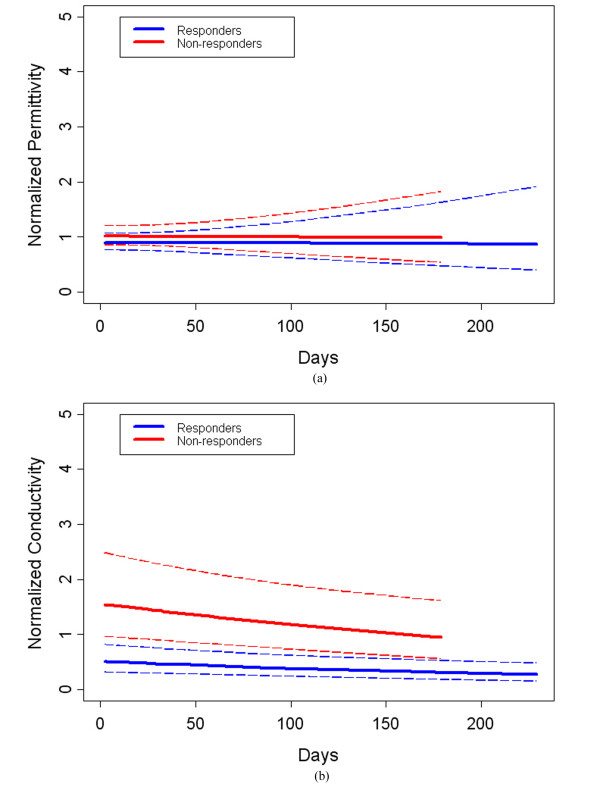
**Fractional change summary for all patients for the 1300 MHz permittivity and conductivity ROI:Background ratios over the full course of treatments**. Dashed lines indicate the 95% confidence intervals for the Responder (in blue) and Non-responder (in red) curves for permittivity in (a) and conductivity in (b). ROI, region of interest.

We studied the normalized ROI:background outcome ratios with respect to baseline as a function of time after initiation of treatment for all eight subjects (four complete response, four incomplete (non)-response). Figure 9 shows a summary of the data points for all eight patients. We applied a linear mixed model with random intercept (subject) and slope effects to analyze both permittivity and conductivity separately. We used the restricted maximum likelihood (REML) method to estimate coefficients. Covariates included in the model were group (1 = complete responder and 0 = non-responder), days from baseline, and interactions between group and days from baseline. Days from baseline was treated as a continuous variable. SAS 9.2 (SAS Institute Inc., Cary, NC, USA) was used to conduct all statistical analyses. Two-side significance level was set at 5%. In order to ensure that the estimated upper and lower value bounds be positive, all statistical analyses were based on the natural log transformed normalized outcome and then transformed back for figure presentation. Figures 10a and 10b show the permittivity and conductivity plots from the fitted analysis curves for the complete-responder and incomplete (non)-responder groups.

The mean of the normalized permittivity ROI:background ratio for complete responders was not significantly different from that for incomplete-responders (*P*-value = 0.68) over the full treatment intervals. However, the corresponding normalized mean conductivity for the complete responders was significantly different from that for non-responders (*P*-value = 0.004). There were no significant time or interaction effects for either dielectric property in the study. We investigated whether other covariates, including body mass index (BMI), age and breast density (we grouped fatty and scattered density breasts into a single low density category, and heterogeneously dense and extremely dense breasts into one high density group, respectively) influenced the results. For both permittivity and conductivity, the effect of these factors was not significant (*P*-values for the three variables were 0.84, 0.36 and 0.32 for permittivity, respectively, and 0.41, 0.58 and 0.07 for conductivity, respectively). We also analyzed the interaction between the group and days from baseline variables and found no association (*P*-values of 0.14 and 0.28 for the permittivity and conductivity, respectively). These results are consistent with our prior experience in which conductivity was found to have a higher level of correlation with tumor presence than the corresponding permittivity values and would be expected to be the superior measure of tumor progression/regression [25]. In addition, the normalized conductivity measure had a high level of association with complete tumor response at 30 days (*P*-value = 0.002) - a finding that could be clinically important because it would provide valuable early-stage information on tumor progression and whether the current therapy regimen should be continued or modified.

**Figure 11:**
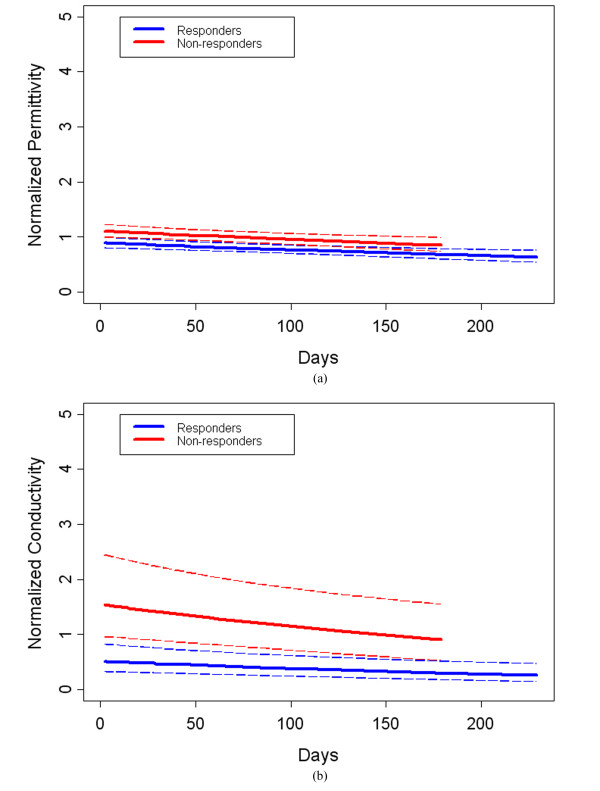
**Fractional change summary for all patients for the 1300 MHz permittivity and conductivity ROI:Background ratios over the full course of treatment with outliers removed**. Dashed lines indicate the 95% confidence intervals for the Responder (in blue) and Non-responder (in red) curves for permittivity in (a) and conductivity in (b). ROI, region of interest.

Finally, we assessed whether outliers may have influenced these results. After removing the data point from Patient #2 on day 104 for both permittivity and conductivity, we compared outcomes between the two groups with the same statistical model described above. In this instance, significant differences resulted between the responder and non-responder groups for both permittivity and conductivity; the *P*-values were 0.0024 and 0.0019, respectively (Figures 11a and 11b). This outcome confirms our hypothesis that the conductivity is statistically significant; however, the permittivity becomes statistically significant when the data point is removed. While likely caused by the fact that the study is relatively small, the property trajectories in the two groups (responders versus non-responders) can be very different. For the complete responders, tumor is destroyed in all instances and, based on limited data from dielectric probe studies, tissue electrical properties trend downwards after successful treatment. However, the tissue characteristics of non-responders are likely to be more variable because in some cases the tissue properties may stay the same or be similar whereas in others tumor progression is likely to yield even higher dielectric properties over time. As a result, the data point removed from Patient #2 may not be an actual outlier, but rather indicative of a more complex tissue behavior that the non-response might be causing.

## Conclusions

We have conducted a preliminary investigation using MT to monitor the progression of breast cancer response to neoadjuvant chemotherapy. Early indications show that the images are repeatable and sensitive to drug-induced breast changes. Of particular interest is the response at one month after the start of treatment where analysis of the conductivity in the ROI was found to distinguish statistically between the complete and incomplete (non)-responding patient groups. Early prediction of treatment success (or lack thereof) could provide useful information to medical oncologists with respect to altering treatment when a patient is not responding to the initial therapy. MT could be performed at numerous stages during treatment at relatively low cost and may be particularly attractive for this type of longitudinal monitoring where the conventional imaging alternatives (for example, MR; FDG PET) are less feasible because of cost and inconvenience.

## Abbreviations

ER: estrogen receptor; FDG PET: fluorodeoxyglucose positron emission tomography; H & E: hematoxylin and eosin; HER2: human epidermal growth factor receptor-2; MT: microwave tomography; NCT: neoadjuvant chemotherapy; PR: progesterone receptor; PT: paclitaxel and traztuzumab; ROIs: regions of interest.

## Competing interests

Drs. Meaney and Paulsen declare that they are co-founders of Microwave Imaging System Technologies, Inc. (MIST) which does have a financial interest in developing microwave breast imaging equipment. However, MIST did not sponsor this effort related to microwave tomography for chemotherapy monitoring. The remaining authors declare that they have no competing interests.

## Authors' contributions

PM supervised all aspects of the microwave tomography data acquisition, image reconstruction and data analysis and contributed to the manuscript preparation. PK designed the study, recruited volunteers, supervised the patients' treatment protocols and contributed to the manuscript preparation. LM coordinated the oncological aspects of the study and contributed to the manuscript preparation. MC interpreted the conventional modality images including MR. SP supervised the interpretation of all conventional modality images including MR. WW supervised the histopathologic preparation and assessment of the breast tissue samples after mastectomy. GS participated in study design and contributed patients to the study. RD interpreted the conventional modality images including MR. TT designed and supervised the statistical analysis. ZL performed the statistical analysis. SG organized all clinical and microwave data and performed the microwave image reconstructions. MF coordinated the multi-modality imaging sessions and designed and optimized the data acquisition software. TZ performed the microwave examinations, designed some hardware and was involved in the image reconstruction process. NE performed the microwave examinations, designed some microwave electronics and was involved in the clinical data organization. KD supervised all aspects of the microwave tomography and coordination with the Radiology, Oncology and Pathology Departments, assisted in the study design and contributed to the manuscript preparation. All authors read and approved the final manuscript.

## References

[B1] Esserman L Neoadjuvant chemotherapy for primary breast cancer: lessons learned and opportunities to optimize therapy Ann Surg Oncol 2004 11 3S 8S 10.1007/BF02524789 15015703

[B2] Fisher B Brown A Mamounas E Wieand S Robidoux A Margolese RG Cruz AB Jr Fisher ER Wickerham DL Wolmark N DeCillis A Hoehn JL Lees AW Dimitrov NV Effect of preoperative chemotherapy on local-regional disease in women with operable breast cancer: findings from National Surgical Adjuvant Breast and Bowel Project B-18 J Clin Oncol 1997 15 2483 2493 9215816 10.1200/JCO.1997.15.7.2483

[B3] Partridge SC Gibbs JE Lu Y Esserman LJ Tripathy D Wolverton DS Rugo HS Hwang ES Ewing CA Hylton NM MRI measurements of breast tumor volume predict response to neoadjuvant chemotherapy and recurrence-free survival Am J Roentgenol 2005 184 1774 1781 10.2214/ajr.184.6.01841774 15908529

[B4] American College of Radiology Imaging Network ACRIN Protocol 6657: contrast-enhanced breast MRI for evaluation of patients undergoing neoadjuvant treatment for locally-advanced breast cancer 2007 http://www.acrin.org/TabID/147/Default.aspx

[B5] Rousseau C Devillers A Sagan C Ferrer L Bridji B Campion L Ricaud M Bourbouloux E Doutriaux I Clouet M Berton-Rigaud D Bouriel C Delecroix V Garin E Rouquette S Resche I Kerbrat P Chatal JF Campone M Monitoring of early response to neoadjuvant chemotherapy in stage II and III breast cancer by [18F]fluorodeoxyglucose positron emission tomography J Clin Oncol 2006 24 5366 5372 10.1200/JCO.2006.05.7406 17088570

[B6] Manton DJ Chaturvedi A Hubbard A Lind MJ Lowry M Maraveyas A Pickles MD Tozer DJ Turnbull LW Neoadjuvant chemotherapy in breast cancer: early response prediction with quantitative MR imaging and spectroscopy Br J Cancer 2006 94 427 435 10.1038/sj.bjc.6602948 16465174 PMC2361138

[B7] Tripathy D Jiang L Rao N McColl R Xie X Weatherall P Ding L Mason R Blood oxygen level dependent (BOLD) contrast MRI and breast cancer chemotherapy response Proc Am Soc Clin Oncol Meet 2006

[B8] Meisamy S Bolan PJ Baker EH Bliss RL Gulbahce E Everson LI Nelson MT Emory TH Tuttle TM Yee D Garwood M Neoadjuvant chemotherapy of locally advanced breast cancer: predicting response with in vivo (1)H MR spectroscopy--a pilot study at 4 T Radiol 2004 233 424 431 10.1148/radiol.2332031285 15516615

[B9] Bonadonna G Valagussa P Brambilla C Ferrari L Molinterni A Terenziani M Zambetti M Primary chemotherapy in operable breast cancer: eight years experience at the Milan Cancer Institute J Clin Oncol 1998 16 93 100 9440728 10.1200/JCO.1998.16.1.93

[B10] Huber S Medl M Helbich T Taucher S Wagner T Rudas M Zuna I Delorme S Locally advanced breast carcinoma: computer assisted semiquantitative analysis of color Doppler ultrasonography in the evaluation of tumor response to neoadjuvant chemotherapy (work in progress) J Ultrasound Med 2000 19 601 607 10972556 10.7863/jum.2000.19.9.601

[B11] Cerussi A Hsiang D Shah N Mehta R Durkin A Butler J Tromberg BJ Predicting response to breast cancer neoadjuvant chemotherapy using diffuse optical spectroscopy Proc Natl Acad Sci USA 2007 104 4014 4019 10.1073/pnas.0611058104 17360469 PMC1805697

[B12] Jiang S Pogue BW Carpenter CM Poplack SP Wells WA Kogel CA Forero JA Muffly LS Schwartz GN Paulsen KD Kaufman PA Evaluation of breast tumor response to neoadjuvant chemotherapy with tomographic diffuse optical spectroscopy: case studies of tumor region-of-interest changes Radiol 2009 252 551 560 10.1148/radiol.2522081202 PMC2753781 19508985

[B13] Dunnwald LK Gralow JR Ellis GK Livingston RB Linden HM Lawton TJ Barlow WE Schubert EK Mankoff DA Residual tumor uptake of [99mTc]-sestamibi after neoadjuvant chemotherapy for locally advanced breast carcinoma predicts survival Cancer 2005 103 680 688 10.1002/cncr.20831 15637688

[B14] Craddock IJ Nilavalan R Leendertz J Preece A Benjamin R Experimental investigation of real aperture synthetically organised radar for breast cancer detection Dig IEEE Antennas Propag Soc Int Symp 2005 1B 179 182

[B15] Fear EC Li X Hagness SC Stuchly MA Confocal microwave imaging for breast cancer detection: localization of tumors in three dimensions IEEE Trans Biomed Eng 2002 49 812 822 10.1109/TBME.2002.800759 12148820

[B16] Yun X Fear EC Johnston RH Compact antenna for radar-based breast cancer detection IEEE Trans Antennas Propag 2005 53 2374 2380

[B17] Xu L Bond EJ van Veen BD Hagness SC An overview of ultra-wideband microwave imaging via space-time beamforming for early-stage breast cancer detection IEEE Antenn Propag Mag 2005 47 19 34

[B18] Davis SK Tandradinata H Hagness SC van Veen BD Ultrawideband microwave breast cancer detection: a detection-theoretic approach using the generalized likelihood ratio test IEEE Trans Biomed Eng 2005 52 1237 1250 10.1109/TBME.2005.847528 16041987

[B19] Bardati F Iudicello S Tognolatti P A Microwave radiometer for diagnosis of breast malignancy Int Conf Electromagnetics Adv App 2007 1014 1017

[B20] Carr KL Microwave radiometry: its importance to the detection of cancer IEEE Trans Microwave Theory Tech 1989 37 1862 1869 10.1109/22.44095

[B21] Carr KL Cevasco P Dunlea P Shaeffer J Radiometric sensing: an adjuvant to mammography to determine breast biopsy IEEE MTT-S Int Microw Symp Dig 2000 2 929 932

[B22] Ciocan R Jiang H Model-based microwave image reconstruction: simulations and experiments Med Phys 2004 31 3231 3241 10.1118/1.1812871 15651607

[B23] Meaney PM Fanning MW Li D Poplack SP Paulsen KD A clinical prototype for active microwave imaging of the breast IEEE Trans Microwave Theory Tech 2000 48 1841 1853 10.1109/22.883861

[B24] Poplack SP Paulsen KD Hartov A Meaney PM Pogue B Tosteson T Grove M Soho S Wells W Electromagnetic breast imaging - average tissue property values in women with negative clinical findings Radiol 2004 231 571 580 10.1148/radiol.2312030606 15128998

[B25] Poplack SP Paulsen KD Hartov A Meaney PM Pogue B Tosteson T Grove M Soho S Wells W Electromagnetic breast imaging - pilot results in women with abnormal mammography Radiol 2007 243 350 359 10.1148/radiol.2432060286 17400760

[B26] Meaney PM Fanning MW Raynolds T Fox CJ Fang Q Kogel CA Poplack SP Paulsen KD Initial clinical experience with microwave breast imaging in women with normal mammography Acad Radiol 2007 14 207 218 10.1016/j.acra.2006.10.016 17236994 PMC1832118

[B27] Fang Q Meaney PM Paulsen KD Microwave image reconstruction of tissue property dispersion characteristics utilizing multiple frequency information IEEE Trans Microwave Theory Tech 2004 52 1866 1875 10.1109/TMTT.2004.832014

[B28] Paulsen KD Meaney PM Compensation for nonactive array element effects in a microwave imaging system: part I - forward solution vs. measured data comparison IEEE Trans Med Imag 1999 18 496 507 10.1109/42.781015 10463128

[B29] Paulsen KD Meaney PM Moskowitz MJ Sullivan JM Jr A dual mesh scheme for finite element based reconstruction algorithms IEEE Trans Med Imag 1995 14 504 514 10.1109/42.414616 18215855

[B30] Meaney PM Paulsen KD Ryan TP Two-dimensional hybrid element image reconstruction for TM illumination IEEE Trans Antennas Propag 1995 43 239 247 10.1109/8.371992

[B31] Lazebnik M Popovic D McCartney L Watkins CB Lindstrom MJ Harter J Sewall S Ogilvie T Magliocco A Breslin TM Temple W Mew D Booske JH Okoniewski M Hagness SC A large-scale study of the ultrawideband microwave dielectric properties of normal, benign and malignant breast tissues obtained from cancer surgeries Phys Med Biol 2007 52 6093 6115 10.1088/0031-9155/52/20/002 17921574

[B32] Pinder SE Provenzano E Earl H Ellis IO Laboratory handling and histology reporting of breast specimens from patients who have received neo-adjuvant chemotherapy Histopathology 2007 50 409 417 10.1111/j.1365-2559.2006.02419.x 17448015

[B33] Fox CJ Meaney PM Shubitidze F Potwin L Paulsen KD Characterization of a monopole antenna in a lossy medium for microwave breast computed tomography Int J Ant and Propag 2008 2008 580782 10.1155/2008/580782 PMC2860330 20428324

